# Short- and Long-term survival prediction in patients with acute type A aortic dissection undergoing open surgery

**DOI:** 10.1186/s13019-024-02687-x

**Published:** 2024-04-02

**Authors:** Yusanjan Matniyaz, Yuan-Xi Luo, Yi Jiang, Ke-Yin Zhang, Wen-Zhe Wang, Tuo Pan, Dong-Jin Wang, Yun-Xing Xue

**Affiliations:** 1 Department of Cardiac Surgery, Affiliated Drum Tower Hospital, Medical School of Nanjing University, Number 321 Zhongshan Road, Nanjing, Jiangsu 210008 China; 2 Department of Cardiac Surgery, Nanjing Drum Tower Hospital, Affiliated Hospital of Medical School, Nanjing University, Number 321 Zhongshan Road, Nanjing, Jiangsu 210008 China; 3 Department of Cardiac Surgery, Affiliated Drum Tower Hospital, Chinese Academy of Medical Sciences & Peking Union Medical College, Number 321 Zhongshan Road, Nanjing, Jiangsu 210008 China; 4 Department of Cardiac Surgery, Nanjing Drum Tower Hospital Clinical College of Nanjing University of Chinese Medicine, Number 321 Zhongshan Road, Nanjing, Jiangsu 210008 China

**Keywords:** Acute type A aortic dissection, Survival prediction, Risk factors, Nomogram

## Abstract

**Background:**

Acute Type A aortic dissection (ATAAD) is a life-threatening cardiovascular disease associated with high mortality rates, where surgical intervention remains the primary life-saving treatment. However, the mortality rate for ATAAD operations continues to be alarmingly high. To address this critical issue, our study aimed to assess the correlation between preoperative laboratory examination, clinical imaging data, and postoperative mortality in ATAAD patients. Additionally, we sought to establish a reliable prediction model for evaluating the risk of postoperative death.

**Methods:**

In this study, a total of 384 patients with acute type A aortic dissection (ATAAD) who were admitted to the emergency department for surgical treatment were included. Based on preoperative laboratory examination and clinical imaging data of ATAAD patients, logistic analysis was used to obtain independent risk factors for postoperative in-hospital death. The survival prediction model was based on cox regression analysis and displayed as a nomogram.

**Results:**

Logistic analysis identified several independent risk factors for postoperative in-hospital death, including Marfan syndrome, previous cardiac surgery history, previous renal dialysis history, direct bilirubin, serum phosphorus, D-dimer, white blood cell, multiple aortic ruptures and age. A survival prediction model based on cox regression analysis was established and presented as a nomogram. The model exhibited good discrimination and significantly improved the prediction of death risk in ATAAD patients.

**Conclusions:**

In this study, we developed a novel survival prediction model for acute type A aortic dissection based on preoperative clinical features. The model demonstrated good discriminatory power and improved accuracy in predicting the risk of death in ATAAD patients undergoing open surgery.

**Supplementary Information:**

The online version contains supplementary material available at 10.1186/s13019-024-02687-x.

## Backgroud

Acute type A aortic dissection (ATAAD) is a severe cardiovascular condition characterized by a tear in the inner lining of the aorta, posing a life-threatening risk to patients. Surgical intervention is the primary life-saving treatment approach for ATAAD. However, During the last decade, in-hospital mortality was reported to be 22% according to contemporary studies [1–3].

**Fig. 1:**
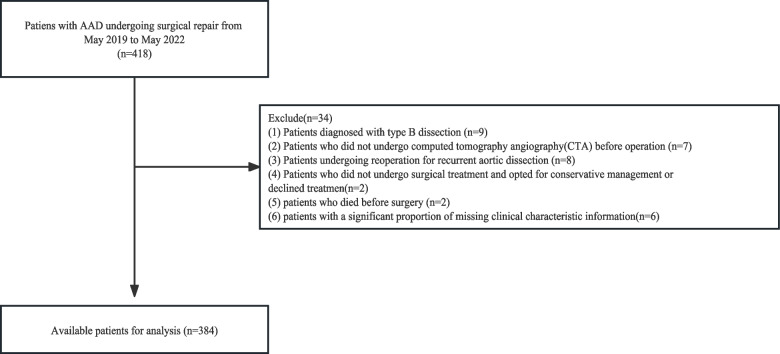
Flowchart of participant selection

Despite advancements in medical care, several challenges contribute to the elevated mortality rate in ATAAD cases. These challenges include difficulties in timely surgical screening [4], delayed diagnosis, limited access to specialized care, and the intricate nature of surgical procedures. Consequently, there is a pressing need to identify key prognostic factors that are linked to these challenges and develop targeted interventions to address them effectively. Current prognostic studies on mortality in ATAAD patients have demonstrated suboptimal performance and significant variability [5]. This highlights the necessity for significant efforts to enhance the utilization of predictive models and investigations into prognostic factors for this patient population. The flowchart is shown in Fig. 1.

In light of the aforementioned issues, our objective was to conduct a comprehensive analysis incorporating preoperative clinical data, serum markers, and imaging studies [6]. By taking this multifaceted approach, we aimed to identify independent risk factors associated with mortality in hospitalized ATAAD patients. The ultimate goal was to develop a reliable nomogram that can accurately predict survival outcomes in ATAAD patients.

By employing this predictive model, surgeons can significantly improve their ability to assess the risk of early postoperative mortality in patients undergoing acute type A surgery. This, in turn, will aid in formulating effective surgical strategies to enhance patient survival and overall outcomes.

## Methods

### Patients

This study received approval from the ethical committees of Nanjing Drum Tower Hospital (No.2022–157-01). It was not feasible to obtain informed consent from all patients due to the nature of the study. However, since this study posed no risk to the patients involved, the institutional ethics committee waived the requirement for informed consent. The study adhered strictly to the Declaration of Helsinki (seventh revision, 2013) and was conducted under the supervision of the ethics committee. After obtaining approval from the ethical committees, a review was conducted using hospital medical records, nursing records, laboratory data, and surgical databases. This retrospective study enrolled patients diagnosed with acute aortic dissection (AAD) who underwent open surgery at Nanjing Drum Tower Hospital between March 2019 and March 2022. A total of 384 patients were included in our study. The initial data was screened using exclusion criteria, which consisted of the following: (1) patients diagnosed with type B dissection, (2) patients who did not undergo computed tomography angiography (CTA) before operation, (3) patients undergoing reoperation for recurrent aortic dissection, (4) patients who did not undergo surgical treatment and opted for conservative management or declined treatment,(5) patients died before surgery, (6) patients with a significant proportion of missing clinical characteristic information.

### Surgical techniques

After the completion of anesthesia, the thoracic cavity is accessed through a median sternotomy. The axillary artery is then freed, and extracorporeal circulation is established through either the axillary artery or femoral artery, along with the superior and inferior vena cava or unicaval vein. Cardiac arrest is induced. The surgeon examines the aortic root and valves to determine the subsequent aortic root surgery. In our center, we employ the "double vest wrap" technique for root reconstruction surgery [7].

The procedure for aortic root reconstruction is as follows: Firstly, the thrombus in the remaining aortic root dissection is completely removed. A polyester sheet is then cut to match the shape of the dissection and placed between the aortic media and adventitia. Subsequently, a strip-shaped lining, consisting of a Dacron sheet, is positioned on the aortic intima surface. The tape-shaped Dacron sheet, the aortic media, the Dacron sheet within the dissection, and the adventitia are continuously sutured using 5–0 polypropylene sutures to form a new proximal aorta. Next, the avulsed aortic valve is reattached to the wall of the aortic sinus. Finally, the aortic medial layer, the Dacron sheet within the dissection, and the adventitia are reinforced using interrupted sutures of polypropylene sutures along the proximal edge of the dissection.

In patients without root involvement and aortic insufficiency, ascending aortic replacement is typically performed. However, for patients with a dilated aortic arch (≥ 45 mm), a tear located in the aortic arch, or damage to the aortic arch structure, total arch replacement with a frozen elephant trunk technique is generally employed. In cases where the aortic arch is damaged, the options include hemiarch replacement or fenestrated arch stenting [8, 9].

### Data collection

Patient laboratory tests, clinical features, and surgical-related information were extracted from our Hospital Information System (HIS). Follow-up data is collected through telephone interviews. The imaging information was obtained from the hospital's imaging system, and the extraction of CTA information was performed by two experienced radiologists. Complete blood count, comprehensive biochemical profile, coagulation function, cardiac enzymes, B-type natriuretic peptide (BNP), and other relevant indicators are obtained through urgent investigations after emergency admission.

The Inflammation Index was calculated using the following formula:

$$\begin{array}{c}\begin{array}{c}\mathrm{Neutrophil}-\mathrm{to}-\mathrm{lymphocyte}\;\mathrm{ratio}\;(\mathrm{NLR})\;=\;\mathrm{Neutrophil}\;\mathrm{count}\;/\;\mathrm{Lymphocyte}\;\mathrm{count}, \mathrm{Monocyte}-\mathrm{to}-\mathrm{lymphocyte}\;\mathrm{ratio}\;(\mathrm{MLR})\;=\;\mathrm{Monocyte}\;\mathrm{count}\;/\;\mathrm{Lymphocyte}\;\mathrm{count}, \mathrm{Systemic}\;\mathrm{inflammation}\;\mathrm{response}\;\mathrm{index}\;(\mathrm{SIRI})\;=\;\mathrm{Monocyte}\;\mathrm{count}\;\ast\;\mathrm{NLR}, \mathrm{Platelet}-\mathrm{to}-\mathrm{lymphocyte}\;\mathrm{ratio}\;(\mathrm{PLR})\;=\;\mathrm{Platelet}\;\mathrm{count}\;/\;\mathrm{Lymphocyte}\;\mathrm{count},\\\mathrm D-\mathrm{dimer}-\mathrm{to}-\mathrm{lymphocyte}\;\mathrm{ratio}\;(\mathrm D-\mathrm{dimer}/\mathrm L)\;=\;\mathrm D-\mathrm{dimer}\;/\;\mathrm{Lymphocyte}\;\mathrm{count}, \mathrm{Systemic}\;\mathrm{immune}-\mathrm{inflammation}\;\mathrm{index}\;(\mathrm{SII})\;=\;\mathrm{Platelet}\;\mathrm{count}\;\ast\;\mathrm{Neutrophil}\;\mathrm{count}\;/\;\mathrm{Lymphocyte}\;\mathrm{count}, \mathrm{Systemic}\;\mathrm{coagulation}-\mathrm{inflammation}\;\mathrm{index}\;(\mathrm{SCI})\;=\;\mathrm{Platelet}\;\mathrm{count}\;\ast\;\mathrm{Fibrinogen}\;\mathrm{count}\;/\;\mathrm{White}\;\mathrm{blood}\;\mathrm{cell}\;\mathrm{count}\end{array}\end{array}$$

### Statistical analysis

Continuous variables were generally described as mean ± SD or median with interquartile ranges (IQR), while discrete variables were expressed as frequencies (*n*, %). The student t-test was utilized for normally distributed continuous variables, while the Mann–Whitney U nonparametric method was employed for non-normally distributed continuous variables. Categorical data were compared using either the chi-square test or Fisher exact test. Receiver operating characteristic (ROC) curves and Youden indices were used to assess predictive values and cutoff points. Logistic regression and Cox regression analyses were performed to assess the independent risk factors for in-hospital death and postoperative survival, respectively. Statistical significance was considered when the two-tailed p-value was less than 0.05. Postoperative survival analysis was carried out using the Kaplan–Meier (KM) method and the Log rank test. The prediction accuracy of the nomogram was evaluated using the time-dependent receiver operating characteristic curves, with the areas under the curve (AUC) calculated. The calibration curve was utilized to assess the consistency between the predicted survival probability of the nomogram and bootstrap resamples. Additionally, decision curve analysis (DCA) was employed to evaluate the net benefit of the nomogram. We excluded variables with a missing value ratio exceeding 10% and conducted multiple imputation for variables with a missing value ratio below 10%. All statistical analyses were conducted using SPSS version 26.0 (IBM, USA) and R version 4.3.0, with appropriate packages and functions utilized.

## Results

A total of 384 patients undergoing open surgery were included in our retrospective study. Among them, 42 (10.9%) patients died within 30 days of hospitalization. The demographic and social data of the two groups revealed that out of the total patients, 307 (79.9%) patients were males, and the average age of the patients was 56.09 ± 13.48 years. A significant portion of the patients, 287 (74.7%) patients had a history of hypertension. Additionally, 141 (36.7%) patients had smoking history, while 21(5.5%) patients had a history of coronary atherosclerotic heart disease. Aortic valve replacement was performed in 75(19.5%) patients, while partial arch replacement of the aorta was performed in 204 (53.1%) patients. 18 (4.7%) patients underwent coronary artery bypass grafting. The group of patients died exhibited a higher frequency of blood transfusions compared to the group of patients who survived {TPT: death group 168.75 [108.12, 238.75] vs. survival group 100.00 [75.00, 144.38], *P* < 0.001; Platelets: death group 2.00 [1.00, 3.00] vs. survival Group 1.00 [1.00, 2.00], *P* < 0.001; TTCF: Death group 12.12 [8.94, 15.00] vs. Survival group 9.50 [8.00, 13.00], *P* = 0.007; TTRBC LR: Death group 15.50 [9.62, 26.12] vs. Survival group 8.50 [6.00, 12.50], *P* < 0.001;}. Moreover, the death group had a longer postoperative invasive ventilator time compared to the survival group {death group: 103.50 [56.00, 192.75] vs. survival group: 25.00 [16.00, 66.00]}. However, there were no significant differences between the deceased and surviving groups in terms of calculated inflammatory indices (SIRI, NLR, MLR, PLR, SII, SCI). Regarding imaging data, the rate of patients with multiple tears in the survival group was significantly higher than in the death group {survival group: 69 cases (20.2%) vs. death group: 1 case (2.4%), *p* = 0.009}. Additionally, the death group had a higher incidence of cumulative mesenteric artery involvement in dissection compared to the survival group {survival group: 31 cases (9.1%) vs. death group: 10 cases (23.8%)}. The baseline data characteristics of the two groups of patients are presented in Table 1. The comparison results of the baseline data of two groups of patients over 30 days are shown in the more detail [see Additional file 1].

**Table 1:** Baseline characteristics

Variables	Overall(*n* = 384)	Survivor (*n* = 342)	Non-survivor (*n* = 42)	*P* value
Gender (male, %)	307 (79.9)	273 (79.8)	34 (81.0)	0.930
Age (year)	56.09 (13.48)	55.82 (13.46)	58.26 (13.64)	0.268
BMI (kg/m2)	25.72 (3.45)	25.79 (3.46)	25.13 (3.28)	0.240
Hospitalization days (mean (SD))	16.71 (9.44)	17.79 (9.15)	7.98 (6.96)	< 0.001
Time of onset (hour)	34.09 (86.45)	36.99 (91.17)	10.45 (6.30)	0.060
LUL SBP (mmHg)	132.43 (26.36)	132.98 (25.87)	128.00 (30.03)	0.249
LUL DBP (mmHg)	71.15 (17.65)	71.64 (17.30)	67.14 (20.13)	0.119
Heart rate (mean (SD))	81.61 (19.51)	81.61 (19.72)	81.62 (17.96)	0.997
Hypertension history (*n*, %)	287 (74.7)	256 (74.9)	31 (73.8)	0.891
Dialysis history (*n*, %)	8 (2.1)	5 (1.5)	3 (7.1)	0.004
Diabetes history (*n*, %)	15 (3.9)	14 (4.1)	1 (2.4)	0.906
Smoking (*n*, %)	141 (36.7)	121 (35.4)	20 (47.6)	0.167
Alcohol consumption (*n*, %)	95 (24.7)	83 (24.3)	12 (28.6)	0.674
Cerebral infarction history (*n*, %)	26 (6.8)	26 (7.6)	0 (0.0)	0.127
Coronary heart disease (*n*, %)	21 (5.5)	19 (5.6)	2 (4.8)	0.311
Lower Limb numbness (*n*, %)	66 (17.2)	57 (16.7)	9 (21.4)	0.579
**Preoperative serological results**				
WBC (10^9/L)	11.60 [8.80, 13.83]	11.45 [8.70, 13.78]	12.60 [10.95, 14.80]	0.045
Neutrophil count (%)	9.90 [7.40, 12.20]	9.70 [7.23, 12.10]	10.55 [8.18, 12.95]	0.127
Lymphocyte count (%)	0.90 [0.60, 1.20]	0.90 [0.60, 1.20]	0.95 [0.62, 1.10]	0.710
Monocytes count (%)	0.60 [0.40, 0.90]	0.60 [0.40, 0.90]	0.75 [0.50, 0.90]	0.109
Eosinophil count (%)	0.01 [0.00, 0.02]	0.01 [0.00, 0.02]	0.00 [0.00, 0.01]	0.282
Basophil count (%)	0.01 [0.01, 0.03]	0.01 [0.01, 0.03]	0.02 [0.01, 0.03]	0.324
PLT (10^9/L)	142.00 [113.00, 186.25]	141.50 [113.00, 185.75]	146.00 [117.25, 186.50]	0.950
BNP (pg/ml)	58.50 [24.20, 154.00]	56.20 [24.20, 143.75]	71.20 [26.55, 189.00]	0.299
ALT (U/L)	29.95 [22.00, 51.25]	29.00 [21.00, 48.75]	39.00 [27.25, 59.50]	0.013
LDH (U/L)	392.50 [269.00, 534.75]	386.00 [263.25, 518.50]	472.00 [285.50, 663.50]	0.030
Total bile acids (umol/L)	2.00 [1.00, 5.40]	1.80 [0.90, 4.70]	3.65 [1.42, 13.23]	0.002
Adenosine deaminase (U/L)	10.80 [9.00, 14.20]	10.80 [8.90, 14.00]	11.50 [10.38, 19.35]	0.027
Urea (mmol/L)	7.00 [5.70, 9.00]	6.90 [5.70, 9.00]	8.15 [5.40, 8.80]	0.704
Creatinine (umol/L)	81.00 [63.77, 110.88]	78.05 [62.15, 105.25]	115.50 [77.18, 193.07]	< 0.001
Uric acid (umol/L))	384.50 [311.00, 463.25]	376.00 [309.50, 453.75]	466.50 [360.25, 545.00]	0.002
TG (mmol/L)	1.11 [0.77, 1.75]	1.08 [0.79, 1.64]	1.25 [0.73, 2.18]	0.375
Phosphorus (mmol/L))	1.12 [0.93, 1.34]	1.10 [0.91, 1.31]	1.23 [1.04, 1.55]	0.010
CRP (mg/L)	8.10 [4.00, 33.60]	9.55 [4.10, 34.88]	7.00 [3.50, 18.05]	0.064
eGFR (median [IQR])	87.75 [60.90, 114.73]	89.50 [62.00, 114.90]	69.75 [56.25, 101.30]	0.022
cTn (ug/L)	0.02 [0.01, 0.09]	0.02 [0.01, 0.08]	0.06 [0.02, 0.18]	0.011
PT (s)	12.40 [11.70, 13.60]	12.40 [11.60, 13.60]	12.50 [11.93, 13.60]	0.284
INR (median [IQR])	1.09 [1.02, 1.19]	1.08 [1.02, 1.19]	1.14 [1.05, 1.21]	0.072
APTT (s)	27.50 [25.90, 30.00]	27.45 [25.90, 29.90]	28.20 [26.30, 30.70]	0.259
TT (s)	18.45 [16.90, 20.25]	18.30 [16.80, 20.17]	18.95 [17.72, 20.80]	0.068
Fibrinogen (g/L)	2.20 [1.60, 3.00]	2.20 [1.60, 3.00]	2.05 [1.50, 2.40]	0.030
D dimer (mg/L)	6.30 [3.22, 13.30]	5.81 [3.01, 12.15]	9.15 [6.15, 33.13]	< 0.001
SIRI	6.97 [4.19, 11.77]	6.94 [4.06, 11.73]	7.44 [5.52, 13.41]	0.189
NLR	11.60 [7.00, 18.67]	11.60 [6.89, 18.67]	11.96 [8.35, 16.59]	0.605
MLR	0.75 [0.50, 1.00]	0.75 [0.50, 1.00]	0.82 [0.56, 1.07]	0.390
PLR	170.00 [122.50, 242.98]	170.56 [120.21, 243.65]	169.28 [136.05, 199.00]	0.807
SII	1668.22 [1004.35, 2631.52]	1641.00 [989.40, 2628.30]	1878.36 [1117.73, 2709.18]	0.710
Dimer(mg/L)	7.82 [3.18, 20.76]	7.28 [2.93, 19.46]	12.45 [6.35, 46.98]	0.004
SCI	27.38 [16.80, 47.48]	27.85 [16.85, 50.97]	23.28 [16.24, 32.42]	0.102
**Preoperative imaging results**				
**False lumen type (** ***n*** **, %)**				0.304
Thromboembolic	177 (46.1)	157 (45.9)	20 (47.6)	
Patent Flow	151 (39.3)	138 (40.4)	13 (31.0)	
Partially Thromboembolic	56 (14.6)	47 (13.7)	9 (21.4)	
Multiple tears (*n*, %)	70 (18.2)	69 (20.2)	1 (2.4)	0.009
Involvement of iliac arteries (*n*, %)	97 (25.3)	85 (24.9)	12 (28.6)	0.738
MA in AD (*n*, %)	41 (10.7)	31 (9.1)	10 (23.8)	0.008
RA in AD (*n*, %)	37 (9.6)	31 (9.1)	6 (14.3)	0.421
FLM SS (*n*, %)	42 (10.9)	36 (10.5)	6 (14.3)	0.635
FLM CS (*n*, %)	307 (79.9)	275 (80.4)	32 (76.2)	0.660
Pericardial effusion (*n*, %)	313 (81.5)	276 (80.7)	37 (88.1)	0.340
Pleural effusion (*n*, %)	52 (13.5)	49 (14.3)	3 (7.1)	0.296
Associated aneurysms (*n*, %)	89 (23.2)	78 (22.8)	11 (26.2)	0.767
True cavity (cm)	2.00 [2.00, 3.00]	2.20 [2.00, 3.00]	2.00 [2.00, 3.00]	0.431
Ascending aortic diameter (cm)	5.00 [4.00, 5.50]	5.00 [4.00, 5.50]	5.00 [5.00, 5.50]	0.051
True cavity total diameter ratio	0.50 [0.36, 0.63]	0.50 [0.36, 0.64]	0.42 [0.36, 0.57]	0.309
False cavity (cm)	2.50 [1.50, 3.00]	2.50 [1.50, 3.00]	2.80 [2.00, 3.50]	0.132
True to false cavity ratio (median [IQR])	1.00 [0.59, 1.75]	1.00 [0.60, 1.75]	0.80 [0.58, 1.52]	0.383
**Operation data**				
**Stent type (** ***n*** **, %)**				0.023
No stent	65 (16.9)	52 (15.2)	13 (31.0)	
TAA + DTA Stent	170 (44.3)	152 (44.4)	18 (42.9)	
DTA Stent	146 (38.0)	136 (39.8)	10 (23.8)	
Valve replacement (*n*, %)	75 (19.5)	65 (19.0)	10 (23.8)	0.593
Aortic arch replacement ((*n*, %)	204 (53.1)	184 (53.8)	20 (47.6)	0.553
CABG (*n*, %)	18 (4.7)	13 (3.8)	5 (11.9)	0.050
Surgical duration (min)	408.57 (102.86)	405.33 (102.04)	434.88 (106.90)	0.079
**Extracorporeal bypass mode (** ***n*** **, %)**				0.040
FA + AxArt + SVC/IVC	196 (51.0)	180 (52.6)	16 (38.1)	
FA + SVC/IVC	120 (31.2)	99 (28.9)	21 (50.0)	
AxArt + SVC/IVC	62 (16.1)	58 (17.0)	4 (9.5)	
AscAo + SVC/IVC	6 (1.6)	5 (1.5)	1 (2.4)	
ECPB (min)	203.97 (61.20)	201.40 (57.08)	224.90 (85.99)	0.019
AXC (min)	148.23 (46.44)	147.63 (46.23)	153.07 (48.40)	0.474
DHCAT (min)	29.26 (12.37)	29.18 (12.47)	29.93 (11.67)	0.712
Postoperative CRRT (*n*, %)	71 (18.5)	46 (13.5)	25 (59.5)	< 0.001
Total plasma transfusion (*10 ml)	100.00 [75.00, 155.00]	100.00 [75.00, 144.38]	168.75 [108.12, 238.75]	< 0.001
Cryoprecipitated coagulation factors (IU)	9.75 [8.00, 13.75]	9.50 [8.00, 13.00]	12.12 [8.94, 15.00]	0.007
Red Blood Cells Suspension (U)	9.00 [6.00, 13.50]	8.50 [6.00, 12.50]	15.50 [9.62, 26.12]	< 0.001
IMV (hour)	31.00 [16.75, 76.25]	25.00 [16.00, 66.00]	103.50 [56.00, 192.75]	< 0.001
ICU (hour)	121.00 [80.50, 182.50]	114.25 [80.50, 179.38]	140.75 [86.12, 228.38]	0.160

*Abbreviations* *:* *BMI* Body mass index, *LUL SBP* Left upper extremity systolic blood pressure, *LUL DBP* Left upper extremity diastolic pressure, *WBC* White blood cell count, *PLT* Platelet count, *BNP* Brain Natriuretic Peptide, *ALT* Glutamate aminotransferase, *LDH* Lactate dehydrogenase, *TG* Triglycerides, *cTn* Troponin, *PT* Prothrombin time, *INR* International standardized ratio, *APTT* Activates partial prothrombin time, *TT* Thrombin time, *SIRI* Systemic inflammation response index, *NLR* Neutrophil-to-lymphocyte ratio, *MLR* Monocyte-to-lymphocyte ratio, *PLR* Platelet-to-lymphocyte ratio, *SII*, Systemic immune-inflammation index, *SCI* Systemic coagulation-inflammation index, *Dimer/L,D* Dimer-to- lymphocyte ratio, *MA* in *AD* Involvement of mesenteric arteries, *RA* in *AD* Involvement of renal arteries, *FLM SS* False lumen morphology (spiderweb sign), *FLM CS* False lumen morphology(crescent sign), *TAA* + *DTA* Stent Full arch and descending aortic stent implantation, *DTA* Stent descending aortic stent, *CABG* Coronary artery bypass graft surgery, *FA* Femoral artery, *SVC* Superior vena cava, *IVC* inferior vena cava, *AxArt* Axillary artery, *AscAo* Ascending aorta, *ECPB* Extracorporeal bypass time, *AXC* Aortic cross clamp time, *DHCAT* Deep hypothermic circulatory arrest time, *IMV* Postoperative invasive ventilator time, *ICU* Duration of stay in the monitoring unit

### Short- term and mid-term outcomes

The short-term and mid-term prognosis results of the patients are presented in Table 2. Within 30 days after surgery, 289 patients (75.3%) experienced at least one postoperative complication during hospitalization. The mortality rate due to gastrointestinal bleeding was higher in the death group compared to the survival group (Death group: 10 cases (23.8%) vs. Survival group: 12 cases (3.5%), *P* < 0.001). The mortality rate after ECMO re-use was higher in the death group compared to the survival group (Death group: 4 patients (9.5%) vs. Survival group: 1 case (0.3%), *P* < 0.001). The postoperative IABP mortality rate was higher in the death group compared to the survival group (Death group: 2 cases (4.8%) vs. Survival group: 0 patients, *P* = 0.004). The postoperative CRRT mortality rate was higher in the death group compared to the survival group (Death group: 25 cases (59.5%) vs. Survival group: 46 cases (13.5%)). During the follow-up period, 47 cases (12.2%) of patients required a second surgical intervention. Additionally, 5 patients (1.3%) underwent a third surgical intervention. Among patients with aortic dissection, 42 patients (10.9%) experienced mortality within 30 days of hospitalization. Within 90 days after surgery, 53 patients (13.8%) experienced mortality, while within 1 year after surgery, 59 patients (15.3%) experienced mortality. There were 67 patients (17.4%) who experienced mortality within 4 years after the operation.

**Table 2:** Postoperative short and mid-term prognosis data

Variables	Alive(*n* = 342)	Death(*n* = 42)	Overall(*n* = 384)	*P* value
Gastrointestinal bleeding (*n*, %)	12 (3.5)	10 (23.8)	22 (5.7)	< 0.001
Perifascial syndrome (*n*, %)	3 (0.9)	1 (2.4)	4 (1.0)	0.921
Cerebral infarction (*n*, %)	24 (7.0)	6 (14.3)	30 (7.8)	0.176
Thoracic exploration (*n*, %)	10 (2.9)	3 (7.1)	13 (3.4)	0.331
Electrical Cardioversion (*n*, %)	4 (1.2)	0 (0.0)	4 (1.0)	0.987
ECMO (*n*, %)	1 (0.3)	4 (9.5)	5 (1.3)	< 0.001
IABP (*n*, %)	0 (0.0)	2 (4.8)	2 (0.5)	0.004
Cerebral ischemia (*n*, %)	2 (0.6)	2 (4.4)	4 (1.0)	0.087
Intracerebral hemorrhage (*n*, %)	2 (0.6)	2 (4.8)	4 (1.0)	0.107
Subarachnoid hemorrhage (*n*, %)	0 (0.0)	2 (4.4)	2 (0.5)	0.005
Endotracheal intubation (*n*, %)	32 (9.4)	20 (47.6)	52 (13.5)	< 0.001
CRRT (*n*, %)	46 (13.5)	25 (59.5)	71 (18.5)	< 0.001
Chest Tube Drainage (*n*, %)	124 (36.6)	11 (26.2)	135 (35.4)	0.247
Limb hemiplegia (*n*, %)	8 (2.3)	0 (0.0)	8 (2.1)	0.668
Second surgery (*n*, %)	42 (12.3)	5 (11.9)	47 (12.2)	0.988
Third surgery (*n*, %)	5 (1.5)	0 (0.0)	5 (1.3)	0.946

### Logistic regression analyses and cox regression analyses

Logistic regression analysis was employed to identify independent risk factors associated with in-hospital mortality within 30 days among patients undergoing open surgery. The results of both univariate and multivariate analyses are presented in Table 3. The results of the multicollinearity diagnosis of multivariate logistic regression are shown in Supplementary file [see Additional file 2]. The results of the multicollinearity diagnosis of multivariate Cox regression are shown in Supplementary file [see Additional file 3].

**Table 3:** Univariate and multivariable logistic regression analyses

**Variables**	**Univariate** **OR (95% CI)**	***P***	**Multivariate** **OR (95% CI)**	***P***
Gender (male)	0.931(0.386,2.009)	0.863		
Age ≥ 58 (year)	2.122(1.112,4.138)	0.024		
Time of onset (hour)	0.971(0.929,0.994)	0.082		
Cardiac surgery history	3.681(0.770,13.839)	0.067		
Nephritis	5.015(1.645,14.022)	0.003	4.494(1.136,17.782)	0.032
BMI (kg/m^2^)	0.944(0.858,1.038)	0.238		
MFS	8.5(0.998,72.432)	0.035	14.016(1.031,190.492)	0.047
Intracerebral hemorrhage	8.5(0.998,72.432)	0.035	12.167(1.080,137.03)	0.043
ECMO again after surgery	18.833(4.761,92.277)	< 0.001		
Postoperative endotracheal intubation	8.807(4.341,17.955)	< 0.001	6.710(2.813,16.007)	< 0.001
Postoperative CRRT	9.463(4.787,19.165)	< 0.001	4.541(1.979,10.421)	< 0.001
WBC ≥ 10.45 (× 10^/L)	2.910(1.371,6.931)	0.009	3.937(1.338,11.579)	0.013
ALT ≥ 33.5(U/L)	2.402(1.255,4.731)	0.009		
ALP ≥ 80 (U/L)	1.003(1.001,1.004)	0.003	1.004(1.001,1.006)	0.001
LDH ≥ 610 (U/L)	3.170(1.549,6.309)	0.001	3.552(1.498,8.424)	0.004
TBIL ≥ 19 (umol/L)	1.025(1.004,1.047)	0.016		
CR ≥ 104 (umol/L)	4.245(2.205,8.356)	< 0.001		
Uric acid (umol/L))	1.004(1.002,1.007)	0.001		
Phosphorus ≥ 1.4 (mmol/L)	3.627(1.813,7.136)	< 0.001		
Fibrinogen (g/L)	0.689(0.490,0.913)	0.021		
D dimer ≥ 4.4 (mg/L)	6.505(2.542,22.065)	< 0.001	3.585(1.095,11.738)	0.035
SIRI	1.018(0.983,1.049)	0.271		
NLR ≥ 7.1	2.308(1.009,6.247)	0.067		
MLR ≥ 0.66	1.369(0.707,2.765)	0.363		
PLR ≥ 118	0.579(0.270,1.158)	0.137		
SII ≥ 1391	1.558(0.748,3.57)	0.260		
Dimer l ≥ 5.56	3.424(1.565,8.606)	0.004		
SCI ≥ 34	0.339(0.143,0.720)	0.008		

*Abbreviations* *:* *BMI* Body mass index, *MFS* Marfan syndrome, *WBC* White blood cell count, *ALT* Glutamate aminotransferase, *LDH* Lactate dehydrogenase, *TBIL* Total bilirubin, *CR* Creatinine, *GFR* Glomerular filtration rate, *SIRI* Systemic inflammation response index, *NLR* Neutrophil-to-lymphocyte ratio, *MLR* Monocyte-to-lymphocyte ratio, *PLR* Platelet-to-lymphocyte ratio, *SII* Systemic immune-inflammation index, *SCI* Systemic coagulation-inflammation index

The univariate logistic regression analysis revealed several potential risk factors for in-hospital mortality within 30 days. These factors included a history of renal nephritis with dialysis, Marfan syndrome, age over 58 years, postoperative cerebral hemorrhage, reusing ECMO postoperatively, reintubation postoperatively, postoperative use of continuous renal replacement therapy (CRRT), preoperative white blood cell count (WBC) ≥ 10.45 (× 10^9/L), alanine transaminase (ALT) ≥ 33.5 (U/L), alkaline phosphatase (ALP) ≥ 80 (U/L), lactate dehydrogenase (LDH) ≥ 610 (U/L), total bilirubin (TBIL) ≥ 19 (umol/L), creatinine (CR) ≥ 104 (umol/L), uric acid, phosphorus ≥ 1.4 (mmol/L), glomerular filtration rate (GFR), fibrinogen, D-dimer ≥ 4.4 (mg/L), Dimer l ≥ 5.56, and SCI ≥ 34.

The multivariate logistic regression analysis demonstrated that the following factors exhibited significant associations with in-hospital mortality within 30 days: history of renal nephritis with dialysis (OR: 4.494; 95% CI: 1.136, 17.782; *p* < 0.05), Marfan syndrome (OR: 14.016; 95% CI: 1.031, 190.492; *p* < 0.05), postoperative cerebral hemorrhage (OR: 12.167; 95% CI: 1.08, 137.03; *p* < 0.05), reintubation postoperatively (OR: 6.710; 95% CI: 2.813, 16.007; *p* < 0.001), postoperative use of CRRT (OR: 4.541; 95% CI: 1.979, 10.421; *p* < 0.001), preoperative WBC ≥ 10.45 (× 10^9/L) (OR: 3.937; 95% CI: 1.338, 11.579; *p* < 0.05), ALP ≥ 80 (U/L) (OR: 1.004; 95% CI: 1.001, 1.006; *p* < 0.05), LDH ≥ 610 (U/L) (OR: 3.552; 95% CI: 1.498, 8.424; *p* < 0.05), and D-dimer ≥ 4.4 (mg/L) (OR: 3.585; 95% CI: 1.095, 11.738; *p* < 0.05).

Cox regression analysis was employed to identify independent risk factors associated with long-term survival after open repair of aortic dissection. Variables with p-values less than 0.05 and variables considered clinically significant were included in the multivariate Cox analysis. The results of the multivariate Cox analysis revealed several independent risk factors for survival in patients undergoing open surgery for aortic dissection. These factors included age ≥ 58 years old, history of cardiovascular surgery, Marfan syndrome, previous history of nephritis and dialysis, WBC ≥ 10.45(× 10^9/L), TBIL, phosphorus ≥ 1.4 mmol/L, D-dimer ≥ 4.4 mg/L, and multiple tears in aortic dissection. These findings highlight the significant association between these factors and the long-term survival outcomes of patients who underwent open surgery for aortic dissection. The results of both univariate and multivariate cox analyses are presented in Table 4.

**Table 4:** Univariate and multivariable COX regression analyses

Variables	Univariate HR (95% CI)	*P*	Multivariate HR (95% CI)	*P*
Age ≥ 58(y)	2.350(1.29–3.865)	0.001	2.659(1.584–4.462)	< 0.001
Cardiac surgery history	2.944(1.070–8.097)	0.036	4.242(1.411–12.756)	0.010
MFS	3.801(0.929–15.552)	0.063	12.042(2.597–55.831)	0.001
Nephritis	4.62(2.205–9.029)	< 0.001	4.917(2.349–10.293)	< 0.001
Preoperative CRRT	16.147(2.181–119.564)	0.006		
WBC ≥ 10.45(× 10*9/L)	2.471(1.369–4.458)	0.003	2.451(1.304–4.607)	0.005
ALT ≥ 33.5(U/L)	2.552(1.545–4.125)	< 0.001		
TBIL (umol/L)	1.024(1.010–1.037)	< 0.001	1.021(1.007–1.035)	0.003
CR ≥ 104(umol/L)	3.562(2.190–5.795)	< 0.001		
Phosphorus ≥ 1.4(mmol/L)	2.918(1.766–4.819)	< 0.001	2.705(1.584–4.621)	< 0.001
Fibrinogen	0.748(0.601–0.930)	0.0009		
D-dimer ≥ 4.4(mg/L)	4.183(2.071–8.450)	< 0.001	3.084(1.498–6.35)	0.002
NLR ≥ 7.1	2.716(1.297–5.689)	0.008		
MLR ≥ 0.66	1.827(1.063–3.140)	0.029		
PLR ≥ 118	0.761(0.453–1.277)	0.301		
SII ≥ 1391	1.662(0.905–3.053)	0.101		
Dimer/l ≥ 5.56	3.008(1.609–5.622)	< 0.001		
SCI ≥ 34	0.406(0.225–0.733)	0.003		
False lumen type	1.322(0.952–1.835)	0.096		
Single tear	1.765(1.154–2.699)	0.009		
Multiple tears	0.339(0.136–0.843)	0.020	0.369(0.145–0.939)	0.036
EoI in AD	1.033(0.944–1.130)	0.483		
IB in AD	0.894(0.778–1.026)	0.111		
Involvement of iliac arteries	0.981(0.559–1.722)	0.946		
MA in AD	1.863(0.975–3.56)	0.060		
RA in AD	1.394(0.665–2.919)	0.379		
FLM SS	1.170(0.559–2.451)	0.677		
FLM CS	0.754(0.430–1.325)	0.327		
Pericardial effusion	2.006(0.916–4.391)	0.082		
Pleural effusion	0.612(0.264–1.416)	0.251		
Associated aneurysms	1.087(0.619–1.909)	0.772		
True cavity	0.906(0.715–1.149)	0.416		
Ascending aortic diameter	1.231(0.952–1.591)	0.112		
True cavity total diameter ratio	0.655(0.211–2.036)	0.465		
False cavity	1.126(0.933–1.358)	0.217		
True to false cavity ratio	0.932(0.835–1.040)	0.207		

*Abbreviations* *:* *MFS* Marfan syndrome, *WBC* White blood cell count, *ALT* Glutamate aminotransferase, *LDH* Lactate dehydrogenase, *TBIL* Total bilirubin, *CR* Creatinine, *GFR* Glomerular filtration rate, *SIRI* Systemic inflammation response index, *NLR* Neutrophil-to-lymphocyte ratio, *MLR* Monocyte-to-lymphocyte ratio, *PLR* Platelet-to-lymphocyte ratio, *SII* Systemic immune-inflammation index, *SCI* Systemic coagulation-inflammation index, *EoI* in *AD* Aortic dissection tear involvement ranges, *IB* in *AD* Involvement of branches in aortic dissection, *MA* in *AD* Involvement of mesenteric arteries, *RA* in *AD* Involvement of renal arteries, *FLM SS* False lumen morphology (spiderweb sign), *FLM CS* False lumen morphology(crescent sign)

### Survival prediction model

**Fig. 2:**
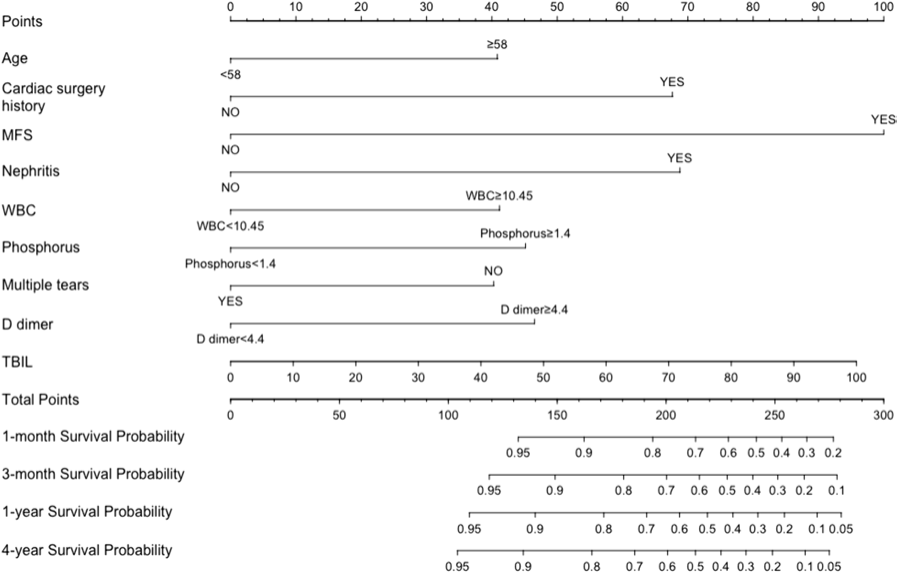
Nomogram for predicting 1-/3- month and 1-/4- year survival of patients with ATAAD undergoing surgical repair

We developed a nomogram utilizing the outcomes of multivariate Cox analysis to prognosticate survival in surgically treated patients with acute aortic dissection. The nomogram incorporates nine noteworthy independent factors, with Marfan syndrome exerting the most substantial influence on survival. Other significant factors comprise a history of previous cardiac surgery, prior renal dialysis, direct bilirubin and serum phosphorus, D-dimer, white blood cell counts, multiple dissection breaks, and age. Each variable is assigned a score on the scoring scale, and the scores are summed to derive a total score, which is then plotted on the corresponding scale. The nomogram encompasses scales that estimate the probability of survival at specific time points, including 1 month, 3 months, 1 year, and 4 years. The nomogram is shown in Fig. 2.

In this, we provide a practical case to demonstrate how to use this model. First, find the corresponding points on the point axis according to the patient's characteristics. For example, a patient is 67 years old, the corresponding points on the points axis are 40; the patient has a history of heart surgery, the corresponding points on the points axis are approximately 67; the patient does not have Marfan syndrome, the points obtained on the points axis are 0; the patient does not have a history of Nephritis, the corresponding points on the points axis are 0; the patient's WBC is 12.8 (× 10*9/L), then it belongs to WBC ≥ 10.45, and the corresponding points on the axis are 40; the patient's phosphorus is 0.73 (mmol/L) and belongs to the ≤ 1.4 category, then the points are 0; the patient has ≥ 2 ruptures, then it belongs to the YES category, and the score on the axis is 0; the patient's D dimer value is 23.94 (mg/L), then it belongs to the ≥ 4.4 category, so the points obtained are approximately 45; the patient's TBIL is 14, the corresponding points on the points axis are 14; then add up all the points to get the total points. In this example, the total points are 40 + 65 + 0 + 0 + 40 + 0 + 0 + 45 + 14 = 204. Next, find the point corresponding to 204 on the total points axis, then draw a line down from the total points obtained until it intersects with the survival probability axis. In this example, the final score is 204, the corresponding 1-month survival rate is approximately 76%, the 3-month survival rate is approximately 70%, the 1-year survival rate is approximately 63%, and the 4-year survival rate is approximately 60%.

**Fig. 3:**
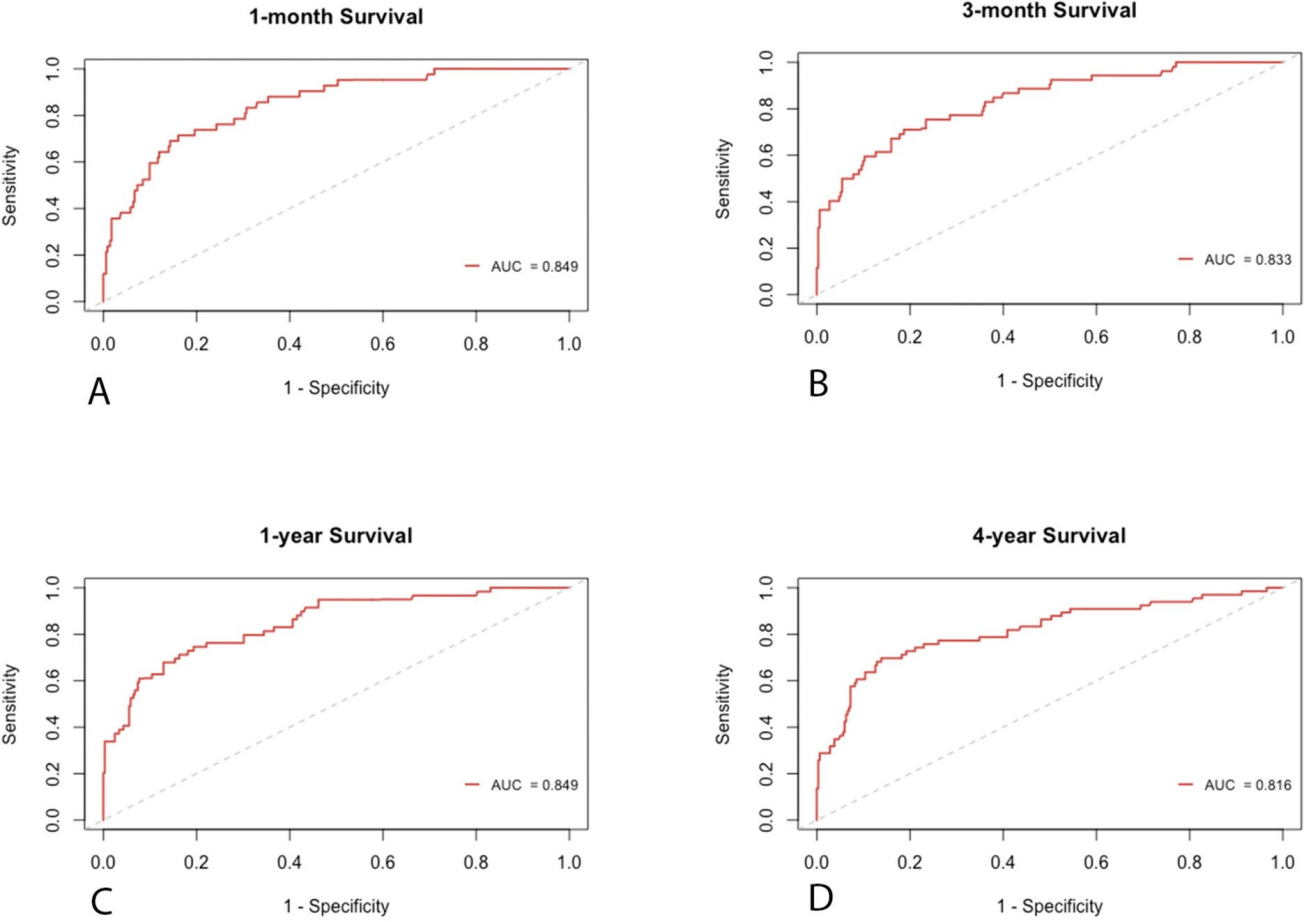
Time-independent ROC curves of the nomogram for 1-/3- month and 1-/4- year survival prediction

**Fig. 4:**
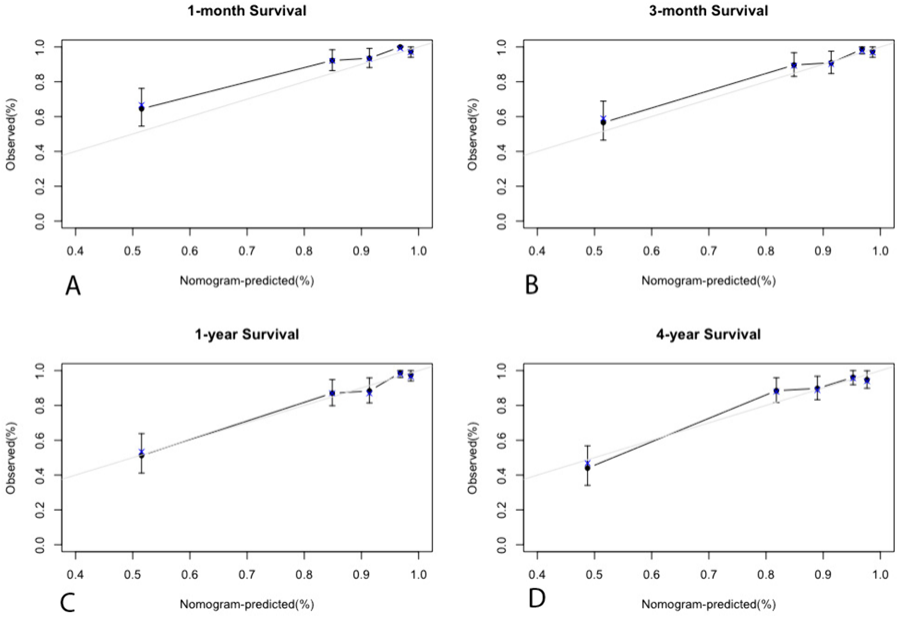
Calibration plots of the nomogram for 1-/3- month and 1-/4- year survival prediction

**Fig. 5:**
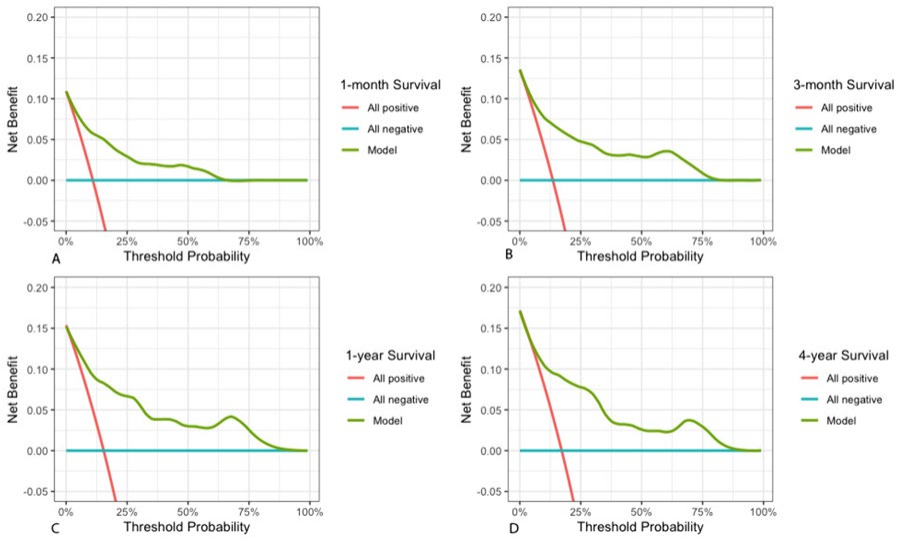
DCA of the nomogram for 1-/3- month and 1-/4- year survival prediction

To assess the predictive capability of the nomogram, we employed time-dependent receiver operating characteristic (ROC) analysis with area under the curve (AUC) (Fig. 3A-D). The results demonstrated the robust predictive power of the nomogram for overall survival at different time points. Specifically, the AUC was 0.849 (95% CI: 0.79–0.91) for the one-month survival probability (Fig. 3A)., 0.833 (95% CI: 0.77–0.89) for the three-month survival probability (Fig. 3B), and 0.849 (95% CI: 0.79–0.90) for the one-year survival probability (Fig. 3C). For the four-year survival probability, the AUC was 0.816 (95% CI: 0.75–0.88) (Fig. 3D). Furthermore, the calibration plot demonstrated excellent concordance between the predictions of the nomogram and the actual observations of overall survival at each time point (Figs. 4A-D and 5). This indicates that the nomogram provides accurate and reliable survival predictions. To evaluate the clinical applic ability of the model, we employed Decision Curve Analysis (DCA) curves. The findings indicate that the model is effective in predicting the one- and three-month as well as the one- and four-year survival probabilities in patients with acute type A aortic dissection (ATAAD) who undergo surgical repair.

#### Robustness of the final model

The robustness of the final model was examined by repeatedly refitting the model to 300 differently sampled training and test sets (ratio 80:20) via the bootstrap procedure. The mean AUC is 0.783 with a 95% bootstrap CI of 0.783–0.796.

## Discussion

In this single-center retrospective study, a predictive model was used by clinicians to identify high-risk patients with acute type A aortic dissection (ATAAD) who were scheduled to undergo surgery. We developed a novel ATAAD survival prediction model based on comprehensive preoperative clinical characteristics. The results showed a significant improvement in predicting the risk of death in patients with ATAAD, demonstrating good discriminative power. This predictive model provides invaluable support for clinicians in identifying high-risk ATAAD patients for planned surgery.

In our constructed prediction model, we included nine significant independent risk factors: Marfan syndrome, previous cardiac surgery history, previous renal dialysis history, direct bilirubin level, serum phosphorus level, D-dimer, white blood cell levels, multiple breaches, and age. We found that using fewer variables in other models resulted in less discriminative models, thus emphasizing the importance of including these variables for better prediction. Our model has several advantages over other predictive models. Firstly, it allows for quick acquisition of information upon admission to the emergency department enabling early identification of patients at high risk of in-hospital death. Additionally, most predictive models are biased towards a single variable, potentially leading to a model biased towards a specific patient type. In contrast, our model encompasses clinical characteristics, serology, and imaging, making it applicable to patients with diverse characteristics. Secondly, while most models primarily focus on short-term survival, our model concentrates on mid- and long-term survival.

Unlike predictive models developed by other researchers, our model highlights the significant contributions of Marfan syndrome, previous history of open heart surgery (except for type A aortic dissection), and previous history of renal dialysis [10, 11]. The logistic regression results also demonstrate that Marfan syndrome and previous history of renal dialysis are independent risk factors for death within 30 days in patients with acute aortic dissection undergoing surgical treatment [12–14]. This indicates that surgeons should not solely focus on aortic dissection but also obtain a detailed history of the patient's past medical records.

Inflammation plays a crucial role in the progression of aortic dissection [15–19]. Aortic tissue injury and thrombus formation in the false lumen can trigger an inflammatory response [20]. Previous research found that white blood cells, including neutrophils and macrophages, were found in the torn aortic tissue [21]. Previous studies have indicated that elevated white blood cell counts are associated with increased in-hospital mortality and serve as a risk indicator for adverse events involving the heart, lungs, brain, and systemic conditions [22–24]. Our study reinforces these findings by demonstrating that an increase in white blood cell count is an independent risk factor for death. Similarly, elevated D-dimer levels have been previously associated with in-hospital major adverse events, and our results align with those of previous investigations [25–27]. We also identified direct bilirubin level and serum phosphorus level as predictor variables in our model, which have received limited attention in prior studies. These findings suggest potential directions for future research. Unfortunately, we did not include calculated inflammatory indicators such as NLR, MLR, PLR, SII, D dimer/l, and SCI in our model [27–29]. Although these indicators showed differences in univariate logistic and Cox analyses, they were all excluded in the multivariate analyses, contradicting previous research results.

Our study dedicated considerable effort to analyzing preoperative imaging information of patients with aortic dissection. Unfortunately, we found no significant associations between aortic true-to-false lumen diameter, true-to-false lumen ratio, or anatomically true-to-false lumen ratio and survival. We speculate that this result may be due to the surgical repair of damaged aortic tissue.

However, we did not conduct further investigation into whether the presence of accumulated but unrepaired planes in patients with aortic dissection was associated with subsequent surgical interventions. Looking at the number of breaches in aortic dissection, we discovered that multiple breaches act as a protective factor against the occurrence of the outcome event. This observation is related to the hemodynamics of aortic dissection, as multiple breaches reduce pressure in the true lumen, thereby mitigating tearing and further extension of dissections.

Naturally, this study, like others, has limitations. Firstly, it was a retrospective study, rendering it susceptible to selection bias. Secondly, the study relied on data from a single center, necessitating further testing to determine if the findings are applicable to other centers.

## Limitations

Our study has several limitations. First, it was a retrospective analysis conducted at a single center. Second, despite the sufficient power of our study, the sample size was relatively small. Therefore, further research is needed to validate the conclusions of our study. Third, we used the Robustness method for model validation. Future prospective data from other institutions for external validation of the model may help further test the predictive ability of the line graph and enhance its universality. Fourth, we did not specify the exact cause of death in postoperative patients, which prevented us from linking postoperative death with postoperative complications.

## Conclusions

We have developed a novel survival prediction model based on comprehensive preoperative clinical characteristics information for acute aortic dissection. This model demonstrates a significant improvement in accurately predicting the risk of death in patients with Type A acute aortic dissection (ATAAD). Furthermore, the model exhibits good discriminatory power, allowing clinicians to effectively identify high-risk ATAAD patients who require immediate surgical intervention.

### Supplementary Information

**Supplementary Material 1.**

**Supplementary Material 2.**

**Supplementary Material 3.**

## Data Availability

The datasets generated and/or analysed during the current study are not publicly available due to patients did not signed consents about upload the data but are available from the corresponding author on reasonable request.

## Abbreviations

ATAAD: Acute type A aortic dissection
AAD: Acute aortic dissection
CTA: Computed tomography angiography
HIS: Hospital information system
BNP: B-type natriuretic peptide
NLR: Neutrophil-to-lymphocyte ratio
MLR: Monocyte-to-lymphocyte ratio
SIRI: Systemic inflammation response index
PLR: Platelet-to-lymphocyte ratio
D-dimer/L: D-dimer-to-lymphocyte ratio
SII: Systemic immune-inflammation index
SCI: Systemic coagulation-inflammation index
ECMO: Extracorporeal membrane oxygenation
CRRT: Continuous renal replacement therapy
