# Supplementary material for: Short- and Long-term survival prediction in patients with acute type A aortic dissection undergoing open surgery
Source: J Cardiothorac Surg. 2024 Apr 2;19:171. doi: 10.1186/s13019-024-02687-x (PMC10988835; doi:10.1186/s13019-024-02687-x)
Supplement: Supplementary file 1 — Supplementary Material 1. [file 13019_2024_2687_MOESM1_ESM.docx]

**Table**

**Baseline Characteristics**

| **Variables** | **Overall（n=384)** | **Survivor (n = 318)** | **Non-survivor (n = 66)** | **P value** |
| --- | --- | --- | --- | --- |
| Gender (male, %) | 307 (79.9) | 254 (79.9) | 53 (80.3) | 0.946 |
| Age (year) | 56.09 (13.48) | 55.23 (13.46) | 60.20 (12.95) | 0.006 |
| BMI (kg/m2) | 25.72 (3.45) | 25.83 (3.38) | 25.19 (3.74) | 0.171 |
| Hospitalization days (mean (SD)) | 16.71 (9.44) | 17.34 (8.61) | 13.70 (12.32) | 0.004 |
| Time of onset (hour) | 34.09 (86.45) | 38.36 (94.09) | 13.53 (18.57) | 0.034 |
| LUL SBP (mmHg) | 132.43 (26.36) | 134.00 [118.25, 148.00] | 129.00 [114.25, 146.00] | 0.377 |
| LUL DBP (mmHg) | 71.15 (17.65) | 72.00 [61.00, 83.00] | 64.50 [52.75, 82.00] | 0.042 |
| Heart rate (mean (SD)) | 81.61 (19.51) | 79.00 [69.00, 91.00] | 76.50 [69.25, 90.00] | 0.859 |
| Hypertension history (n, %) | 287 (74.7) | 239 (75.2) | 48 (72.7) | 0.797 |
| Dialysis history (n, %) | 8 (2.1) | 5 (1.5) | 3 (7.1) | 0.004 |
| Diabetes history (n, %) | 15 (3.9) | 14 (4.1) | 1 (2.4) | 0.906 |
| Smoking (n, %) | 141 (36.7) | 113 (35.5) | 28 (42.4) | 0.359 |
| Alcohol consumption (n, %) | 95 (24.7) | 77 (24.2) | 18 (27.3) | 0.713 |
| Cerebral infarction history (n, %) | 26 (6.8) | 26 (7.6) | 0 (0.0) | 0.127 |
| Coronary heart disease (n, %) | 21 (5.5) | 17 (5.3) | 4 (6.1) | 0.946 |
| Lower Limb numbness (n, %) | 66 (17.2) | 56 (17.6) | 10 (15.2) | 0.762 |
| Preoperative serological results |  |  |  |  |
| WBC (10^9/L) | 11.60 [8.80, 13.83] | 11.40 [8.60, 13.80] | 12.60 [10.72, 14.07] | 0.035 |
| Neutrophil count (%) | 9.90 [7.40, 12.20] | 9.55 [7.10, 12.07] | 10.65 [9.20, 12.65] | 0.016 |
| Lymphocyte count (%) | 0.90 [0.60, 1.20] | 0.90 [0.60, 1.20] | 0.85 [0.60, 1.10] | 0.544 |
| Monocytes count (%) | 0.60 [0.40, 0.90] | 0.60 [0.40, 0.90] | 0.70 [0.50, 0.90] | 0.375 |
| Eosinophil count (%) | 0.01 [0.00, 0.02] | 0.01 [0.00, 0.02] | 0.00 [0.00, 0.01] | 0.05 |
| Basophil count (%) | 0.01 [0.01, 0.03] | 0.01 [0.01, 0.03] | 0.02 [0.01, 0.03] | 0.975 |
| PLT (10^9/L) | 142.00 [113.00, 186.25] | 144.50 [113.25, 188.00] | 137.00 [112.00, 171.25] | 0.203 |
| BNP (pg/ml) | 58.50 [24.20, 154.00] | 55.45 [23.60, 133.00] | 89.60 [36.30, 312.75] | 0.011 |
| ALT (U/L) | 29.95 [22.00, 51.25] | 28.00 [21.00, 45.50] | 39.00 [28.00, 66.07] | <0.001 |
| LDH (U/L) | 392.50 [269.00, 534.75] | 370.00 [260.50, 509.00] | 472.00 [287.00, 661.50] | 0.004 |
| Total bile acids (umol/L) | 2.00 [1.00, 5.40] | 1.80 [0.90, 4.50] | 4.70 [1.63, 10.07] | <0.001 |
| Adenosine deaminase (U/L) | 10.80 [9.00, 14.20] | 10.80 [8.90, 13.78] | 11.60 [10.00, 18.48] | 0.008 |
| Urea (mmol/L) | 7.00 [5.70, 9.00] | 6.90 [5.70, 9.00] | 8.20 [5.95, 8.80] | 0.348 |
| Creatinine (umol/L) | 81.00 [63.77, 110.88] | 74.70 [61.08, 102.75] | 108.00 [80.62, 151.38] | <0.001 |
| Uric acid (umol/L)) | 384.50 [311.00, 463.25] | 372.00 [306.50, 453.75] | 431.50 [364.50, 497.00] | 0.001 |
| TG (mmol/L) | 1.11 [0.77, 1.75] | 1.11 [0.79, 1.64] | 1.14 [0.71, 1.77] | 0.885 |
| Phosphorus (mmol/L)) | 1.12 [0.93, 1.34] | 1.10 [0.91, 1.30] | 1.21 [1.02, 1.55] | 0.002 |
| CRP (mg/L) | 8.10 [4.00, 33.60] | 9.50 [4.00, 33.40] | 7.60 [4.03, 32.18] | 0.59 |
| eGFR (median [IQR]) | 87.75 [60.90, 114.73] | 92.65 [66.20, 115.50] | 65.40 [50.40, 89.25] | <0.001 |
| cTn (ug/L) | 0.02 [0.01, 0.09] | 0.02 [0.01, 0.08] | 0.05 [0.02, 0.19] | 0.001 |
| PT (s) | 12.40 [11.70, 13.60] | 12.30 [11.60, 13.50] | 12.80 [11.93, 13.97] | 0.057 |
| INR (median [IQR]) | 1.09 [1.02, 1.19] | 1.08 [1.02, 1.18] | 1.15 [1.06, 1.25] | 0.002 |
| APTT (s) | 27.50 [25.90, 30.00] | 27.40 [25.90, 29.90] | 28.05 [26.15, 30.78] | 0.202 |
| TT (s) | 18.45 [16.90, 20.25] | 18.30 [16.80, 20.00] | 19.05 [17.72, 21.05] | 0.012 |
| Fibrinogen (g/L) | 2.20 [1.60, 3.00] | 2.20 [1.60, 3.00] | 2.05 [1.50, 2.40] | 0.014 |
| D dimer (mg/L) | 6.30 [3.22, 13.30] | 5.66 [2.97, 11.62] | 9.39 [6.01, 29.50] | <0.001 |
| SIRI | 6.97 [4.19, 11.77] | 6.61 [3.91, 11.44] | 9.00 [5.60, 13.19] | 0.018 |
| NLR | 11.60 [7.00, 18.67] | 10.97 [6.84, 18.33] | 13.36 [9.36, 20.75] | 0.026 |
| MLR | 0.75 [0.50, 1.00] | 0.73 [0.50, 1.00] | 0.82 [0.60, 1.12] | 0.099 |
| PLR | 170.00 [122.50, 242.98] | 171.27 [120.21, 243.21] | 167.38 [127.14, 235.46] | 0.815 |
| SII | 1668.22 [1004.35, 2631.52] | 1622.28 [967.28, 2575.43] | 1922.82 [1155.98, 2837.12] | 0.217 |
| Dimer(mg/L) | 7.82 [3.18, 20.76] | 7.00 [2.93, 18.07] | 13.05 [6.65, 40.42] | <0.001 |
| SCI | 27.38 [16.80, 47.48] | 28.04 [16.97, 52.01] | 22.30 [16.24, 32.42] | 0.015 |
| Preoperative imaging results |  |  |  |  |
| False lumen type (n, %) |  |  |  | 0.304 |
| Thromboembolic | 177 (46.1) | 150 (47.2) | 27 (40.9) | 0.118 |
| Patent Flow | 151 (39.3) | 127 (39.9) | 24 (36.4) |  |
| Partially Thromboembolic | 56 (14.6) | 41 (12.9) | 15 (22.7) |  |
| Multiple tears (n, %) | 70 (18.2) | 65 (20.4) | 5 (7.6) | 0.022 |
| Involvement of iliac arteries (n, %) | 97 (25.3) | 81 (25.5) | 16 (24.2) | 0.957 |
| MA in AD (n, %) | 41 (10.7) | 30 (9.4) | 11 (16.7) | 0.13 |
| RA in AD (n, %) | 37 (9.6) | 29 (9.1) | 8 (12.1) | 0.601 |
| FLM SS (n, %) | 42 (10.9) | 34 (10.7) | 8 (12.1) | 0.903 |
| FLM CS (n, %) | 307 (79.9) | 257 (80.8) | 50 (75.8) | 0.444 |
| Pericardial effusion (n, %) | 313 (81.5) | 254 (79.9) | 59 (89.4) | 0.101 |
| Pleural effusion (n, %) | 52 (13.5) | 46 (14.5) | 6 (9.1) | 0.335 |
| Associated aneurysms (n, %) | 89 (23.2) | 73 (23.0) | 16 (24.2) | 0.948 |
| True cavity (cm) | 2.00 [2.00, 3.00] | 2.20 [2.00, 3.00] | 2.00 [2.00, 3.00] | 0.513 |
| Ascending aortic diameter (cm) | 5.00 [4.00, 5.50] | 5.00 [4.00, 5.50] | 5.00 [5.00, 5.50] | 0.039 |
| True cavity total diameter ratio | 0.50 [0.36, 0.63] | 0.50 [0.36, 0.64] | 0.44 [0.36, 0.58] | 0.349 |
| False cavity (cm) | 2.50 [1.50, 3.00] | 2.50 [1.50, 3.00] | 2.80 [2.00, 3.42] | 0.107 |
| True to false cavity ratio (median [IQR]) | 1.00 [0.59, 1.75] | 1.00 [0.60, 1.79] | 0.88 [0.57, 1.50] | 0.38 |
| Operation data |  |  |  |  |
| Stent type (n, %) |  |  |  | 0.181 |
| No stent | 65 (16.9) | 50 (15.7) | 15 (22.7) |  |
| TAA + DTA Stent | 170 (44.3) | 138 (43.4) | 32 (48.5) |  |
| DTA Stent | 146 (38.0) | 128 (40.3) | 18 (27.3) |  |
| Valve replacement (n, %) | 75 (19.5) | 59 (18.6) | 16 (24.2) | 0.373 |
| Aortic arch replacement ((n, %) | 204 (53.1) | 172 (54.1) | 32 (48.5) | 0.487 |
| CABG (n, %) | 18 (4.7) | 10 (3.1) | 8 (12.1) | 0.005 |
| Surgical duration (min) | 408.57 (102.86) | 392.50 [330.00, 470.00] | 420.00 [370.00, 488.75] | 0.024 |
| Extracorporeal bypass mode (n, %) |  |  |  | 0.176 |
| FA+ AxArt +SVC/IVC | 196 (51.0) | 164 (51.6) | 32 (48.5) |  |
| FA+ SVC/IVC | 120 (31.2) | 93 (29.2) | 27 (40.9) |  |
| AxArt+ SVC/IVC | 62 (16.1) | 56 (17.6) | 6 (9.1) |  |
| AscAo+SVC/IVC | 6 (1.6) | 5 (1.6) | 1 (1.5) |  |
| ECPB (min) | 203.97 (61.20) | 190.00 [160.25, 230.00] | 214.00 [168.25, 251.25] | 0.018 |
| AXC (min) | 148.23 (46.44) | 137.50 [113.00, 173.75] | 147.00 [124.00, 182.50] | 0.186 |
| DHCAT (min) | 29.26 (12.37) | 27.00 [20.00, 36.00] | 27.50 [21.25, 36.75] | 0.65 |
| TPT (*10ml) | 100.00 [75.00, 155.00] | 97.50 [72.50, 139.62] | 155.00 [108.12, 235.00] | <0.001 |
| TTCF (median [IQR]) | 9.75 [8.00, 13.75] | 9.50 [8.00, 13.00] | 10.62 [8.56, 15.00] | 0.024 |
| TTRBC LR (median [IQR]) | 9.00 [6.00, 13.50] | 8.00 [5.50, 12.00] | 15.00 [9.62, 25.38] | <0.001 |
| IMV (h) | 31.00 [16.75, 76.25] | 23.00 [16.00, 59.50] | 121.00 [56.00, 268.25] | <0.001 |
| ICU (h) | 121.00 [80.50, 182.50] | 107.25 [77.62, 168.50] | 203.25 [114.38, 415.25] | <0.001 |

**Abbreviations:** BMI, body mass index; LUL SBP, left upper extremity systolic blood pressure; LUL DBP, left upper extremity diastolic pressure; WBC, White blood cell count; PLT, Platelet count; BNP, Brain Natriuretic Peptide; ALT, Glutamate aminotransferase; LDH, lactate dehydrogenase; TG, Triglycerides; cTn, Troponin; PT, Prothrombin time; INR, International standardized ratio; APTT, Activates partial prothrombin time; TT, Thrombin time; SIRI, systemic inflammation response index; NLR, Neutrophil-to-lymphocyte ratio; MLR, Monocyte-to-lymphocyte ratio; PLR, Platelet-to-lymphocyte ratio; SII, systemic immune-inflammation index; SCI, systemic coagulation-inflammation index; Dimer/L,D dimer-to- lymphocyte ratio; MA in AD, Involvement of mesenteric arteries; RA in AD, Involvement of renal arteries; FLM SS, False lumen morphology (spiderweb sign); FLM CS, False lumen morphology(crescent sign); TAA + DTA Stent, Full arch and descending aortic stent implantation; DTA Stent, descending aortic stent; CABG, coronary artery bypass graft surgery; FA, femoral artery; SVC, superior vena cava; IVC, inferior vena cava; AxArt, Axillary artery; AscAo, ascending aorta; ECPB, extracorporeal bypass time; AXC, aortic cross clamp time; DHCAT, deep hypothermic circulatory arrest time; TPT, Total plasma transfusion; TTCF, Cryoprecipitated coagulation factors; TTRBC LR, Red blood cells; IMV, Postoperative invasive ventilator time; ICU, Duration of stay in the monitoring unit;
