# Supplementary material for: Short- and Long-term survival prediction in patients with acute type A aortic dissection undergoing open surgery
Source: J Cardiothorac Surg. 2024 Apr 2;19:171. doi: 10.1186/s13019-024-02687-x (PMC10988835; doi:10.1186/s13019-024-02687-x)
Supplement: Supplementary file 2 — Supplementary Material 2. [file 13019_2024_2687_MOESM2_ESM.docx]

| Variable | Tolerance | VIF |
| --- | --- | --- |
| Gender (male) | 0.780 | 1.281 |
| Age≥58 (year) | 0.790 | 1.265 |
| Time of onset (hour) | 0.765 | 1.307 |
| Cardiac surgery history | 0.814 | 1.228 |
| Nephritis | 0.789 | 1.267 |
| BMI (kg/m²) | 0.828 | 1.208 |
| MFS | 0.932 | 1.073 |
| Intracerebral hemorrhage | 0.941 | 1.063 |
| ECMO again after surgery | 0.780 | 1.283 |
| Postoperative endotracheal intubation | 0.950 | 1.053 |
| Postoperative CRRT | 0.766 | 1.306 |
| WBC≥10.45 (×10^/L) | 0.602 | 1.662 |
| ALT≥33.5(U/L) | 0.733 | 1.364 |
| ALP≥80 (U/L) | 0.758 | 1.318 |
| LDH≥610 (U/L) | 0.718 | 1.394 |
| TBIL≥19 (umol/L) | 0.791 | 1.264 |
| CR≥104 (umol/L) | 0.589 | 1.699 |
| Uric acid (umol/L)) | 0.609 | 1.642 |
| Phosphorus≥1.4 (mmol/L) | 0.800 | 1.251 |
| Fibrinogen (g/L) | 0.615 | 1.627 |
| D dimer≥4.4 (mg/L) | 0.405 | 2.471 |
| SIRI | 0.503 | 1.988 |
| NLR≥7.1 | 0.435 | 2.300 |
| MLR≥0.66 | 0.579 | 1.726 |
| PLR≥118 | 0.637 | 1.570 |
| SII≥1391 | 0.465 | 2.149 |
| Dimer l≥5.56 | 0.344 | 2.905 |
| SCI≥34 | 0.559 | 1.790 |
