# Supplementary material for: Short- and Long-term survival prediction in patients with acute type A aortic dissection undergoing open surgery
Source: J Cardiothorac Surg. 2024 Apr 2;19:171. doi: 10.1186/s13019-024-02687-x (PMC10988835; doi:10.1186/s13019-024-02687-x)
Supplement: Supplementary file 3 — Supplementary Material 3. [file 13019_2024_2687_MOESM3_ESM.docx]

| Variable | Tolerance | | VIF |
| --- | --- | --- | --- |
| Age≥58(y) | | 0.824 | 1.213 |
| Cardiac surgery history | | 0.847 | 1.181 |
| MFS | | 0.894 | 1.118 |
| Nephritis | | 0.803 | 1.245 |
| Preoperative CRRT | | 0.883 | 1.133 |
| WBC≥10.45(×10*9/L) | | 0.646 | 1.549 |
| ALT≥33.5(U/L) | | 0.809 | 1.236 |
| TBIL (umol/L) | | 0.841 | 1.189 |
| CR≥104(umol/L) | | 0.694 | 1.441 |
| Phosphorus≥1.4(mmol/L) | | 0.780 | 1.283 |
| Fibrinogen | | 0.591 | 1.691 |
| D-dimer≥4.4(mg/L) | | 0.394 | 2.541 |
| NLR≥7.1 | | 0.426 | 2.347 |
| MLR≥0.66 | | 0.662 | 1.510 |
| PLR≥118 | | 0.650 | 1.539 |
| SII≥1391 | | 0.454 | 2.201 |
| Dimer/l≥5.56 | | 0.335 | 2.989 |
| SCI≥34 | | 0.528 | 1.893 |
| False lumen type | | 0.853 | 1.172 |
| Single tear | | 0.468 | 2.137 |
| Multiple tears | | 0.450 | 2.223 |
| EoI in AD | | 0.565 | 1.769 |
| IB in AD | | 0.899 | 1.112 |
| Involvement of iliac arteries | | 0.601 | 1.664 |
| MA in AD | | 0.824 | 1.213 |
| RA in AD | | 0.800 | 1.250 |
| FLM SS | | 0.630 | 1.588 |
| FLM CS | | 0.710 | 1.409 |
| Pericardial effusion | | 0.845 | 1.184 |
| Pleural effusion | | 0.818 | 1.223 |
| Associated aneurysms | | 0.737 | 1.356 |
| True cavity | | 0.081 | 12.409 |
| Ascending aortic diameter | | 0.085 | 11.718 |
| True cavity total diameter ratio | | 0.048 | 20.634 |
| False cavity | | 0.027 | 37.313 |
| True to false cavity ratio | | 0.535 | 1.868 |
